# Characterizing the Microbial Consortium L1 Capable of Efficiently Degrading Chlorimuron-Ethyl *via* Metagenome Combining 16S rDNA Sequencing

**DOI:** 10.3389/fmicb.2022.912312

**Published:** 2022-06-23

**Authors:** Xiang Li, Changming Lu, Yumeng Dai, Zhixiong Yu, Wu Gu, Tingting Li, Xinyu Li, Xu Li, Xiujuan Wang, Zhencheng Su, Mingkai Xu, Huiwen Zhang

**Affiliations:** ^1^Key Laboratory of Pollution Ecology and Environmental Engineering, Institute of Applied Ecology, Chinese Academy of Sciences, Shenyang, China; ^2^University of Chinese Academy of Sciences, Beijing, China; ^3^Shenyang Research Institute of Chemical Industry, Shenyang, China

**Keywords:** biodegradation, chlorimuron-ethyl, consortium, metagenomics, network

## Abstract

Excessive application of the herbicide chlorimuron-ethyl (CE) severely harms subsequent crops and poses severe risks to environmental health. Therefore, methods for efficiently decreasing and eliminating CE residues are urgently needed. Microbial consortia show potential for bioremediation due to their strong metabolic complementarity and synthesis. In this study, a microbial consortium entitled L1 was enriched from soil contaminated with CE by a “top-down” synthetic biology strategy. The consortium could degrade 98.04% of 100 mg L^−1^ CE within 6 days. We characterized it from the samples at four time points during the degradation process and a sample without degradation activity *via* metagenome and 16S rDNA sequencing. The results revealed 39 genera in consortium L1, among which *Methyloversatilis* (34.31%), *Starkeya* (28.60%), and *Pseudoxanthomonas* (7.01%) showed relatively high abundances. Temporal succession and the loss of degradability did not alter the diversity and community composition of L1 but changed the community structure. Taxon-functional contribution analysis predicted that glutathione transferase [EC 2.5.1.18], urease [EC 3.5.1.5], and allophanate hydrolase [EC 3.5.1.54] are relevant for the degradation of CE and that *Methyloversatilis, Pseudoxanthomonas, Methylopila, Hyphomicrobium, Stenotrophomonas*, and *Sphingomonas* were the main degrading genera. The degradation pathway of CE by L1 may involve cleavage of the CE carbamide bridge to produce 2-amino-4-chloro-6-methoxypyrimidine and ethyl o-sulfonamide benzoate. The results of network analysis indicated close interactions, cross-feeding, and co-metabolic relationships between strains in the consortium, and most of the above six degrading genera were keystone taxa in the network. Additionally, the degradation of CE by L1 required not only “functional bacteria” with degradation capacity but also “auxiliary bacteria” without degradation capacity but that indirectly facilitate/inhibit the degradation process; however, the abundance of “auxiliary bacteria” should be controlled in an appropriate range. These findings improve the understanding of the synergistic effects of degrading bacterial consortia, which will provide insight for isolating degrading bacterial resources and constructing artificial efficient bacterial consortia. Furthermore, our results provide a new route for pollution control and biodegradation of sulfonylurea herbicides.

## Introduction

The herbicide chlorimuron-ethyl (CE) is extensively used in soybean fields to control annual grass weeds, sedges, and broadleaf weeds (Reddy et al., 1995). However, CE has a half-life of ≈7–70 days in soils and remains present for 2–3 years; CE is phytotoxic toward current and subsequent crops and leads to reductions in the yield and quality of crops (Sharma et al., 2012; Zang et al., 2020a). Moreover, CE residues in the soil can alter the structure of soil microbial communities, reduce soil enzyme activities (Zawoznik and Tomaro, 2005; Zhang et al., 2011), and enter the aquatic environment directly or indirectly, causing water pollution and promoting the growth of harming aquatic organisms (Battaglin et al., 2000; Fenoll et al., 2013). To overcome these issues, new methods and suitable technologies are urgently needed to eliminate CE and its intermediate metabolite residues from the environment. Microbial degradation has great advantages in the restoration of the environment with herbicide residues due to economic, eco-friendly, safety, and no secondary pollution (Singh and Singh, 2016; Jing et al., 2020), and many CE-degrading strains have been isolated, including fungi and bacteria (Zang et al., 2020b). These strains degrade CE under laboratory conditions but show some limitations such as low efficiency, unstable effects, incomplete degradation, and easy repellence by indigenous microorganisms in the remediation of *in situ* contaminated soil. Complex organic pollutants in nature cannot be degraded by a single microbial strain but rather by the syntrophism and metabolism of consortia (Jeon and Madsen, 2013, Vaidya et al., 2018).

Microbial consortia are composite microbial assemblies with stable structure and function and can be cocultivated and metabolized in a specific environment by two or more microorganisms through domestication (VerBerkmoes et al., 2009). Based on the synergistic interaction of different microorganisms in the population, bacterial consortia are more effective than single microbial or enzyme systems for environmental bioremediation (Wanapaisan et al., 2018) due to their better adaptability and tolerance to variable and complicated environments (Xu et al., 2020; Wang et al., 2021). Furthermore, microorganisms in consortia can exchange substances and communicate with each other through complex and efficient metabolic regulation networks and signaling molecules (Zafra et al., 2017; Bai et al., 2022) to coordinate the overall function of the strain and achieve higher degradation efficiency compared with that of a single organism (Bhatia et al., 2018). Therefore, bacterial consortia are good models for studying the interactions between bacterial populations during bioremediation (Desai et al., 2010) and analyzing the network relationships among bacterial communities, metabolic exchange, and signal transmission. Moreover, the consortia are a resource library for cultivated functional bacteria and a powerful tool for evaluating the potential of viable but non-culturable bacteria and for determining the function of many unknown genes (Wintermute and Silver, 2010). Therefore, studies of the microbial ecology of the bacterial consortia are essential for understanding their roles and niches in the degradation process and for optimizing their performance (Eze et al., 2021).

Many bacterial consortia have been reported to degrade herbicides such as linuron (Dejonghe et al., 2003; Zhang et al., 2018), diuron (Ellegaard-Jensen et al., 2014), atrazine (Xu et al., 2019), metribuzin (Wahla et al., 2019), bispyribac sodium (Ahmad et al., 2018), and CE (Li et al., 2017). These consortia showed significantly higher degradation efficiency than a single microorganism, indicating that synergy among the strains can improve metabolic efficiency. The natural microbial consortium for herbicide degradation has not been widely examined, and research on artificial microbial consortia has only focused on their degradation efficiency. Studies of the community diversity, structure, and functional interactions of natural microbial consortiums can reveal the co-metabolism relationship and regulation mechanism during degradation and provide guidelines for constructing a high-efficiency artificial microbial consortium using synthetic biology methods.

In this study, we enriched a natural microbial consortia L1 (MC-L1) from CE-contaminated environments using the “top-down” strategy (Liang et al., 2022). To characterize this consortium, we analyzed the bacterial diversity, structure, function, pathway, and interactions of MC-L1 during the degradation process using 16S rRNA high-throughput sequencing and metagenomic sequencing. We predicted the possible pathways and keystone taxa related to CE degradation. Our results will greatly improve the understanding of degrading bacterial consortia and provide a foundation for applying bacterial consortia to herbicide residue elimination and environmental health remediation.

## Materials and Methods

### Chemicals and Cultural Media

Chlorimuron-ethyl (purity ≥98.0%) was purchased from Acmec biochemical Co., Shanghai, China. All other chemicals, analytical grade or better, were purchased from Sinopharm Chemical Reagent Co., Ltd., China.

The composition of the inorganic salt medium was as follows (g/L): 2.0 g NaNO_3_, 2.0 g KH_2_PO_4_, 0.125 g MgSO_4_·7H_2_O, 0.5 g NaCl, 0.02 g FeSO_4_·7H_2_O. The pH of the medium was adjusted to 7.0.

### Sample Collection and Degradation Capacity Determination

The soil sample was collected from a pesticide factory in Nanjing, Jiangsu, China and then was applied to enrich the CE degrading bacterial consortium. The experiment used standard successive enrichment culture procedures using 100 mg L^−1^ CE as the sole carbon and energy source. The enriched consortium has been maintained by weekly transfers in the inorganic salt medium. After 10 subcultures, a bacterial consortium named L1 with a stable degradation rate was obtained. To further determine the degradation capacity, MC-L1 was transferred into 100 ml of inorganic salt medium (pH = 7.0) containing 100 mg L^−1^ of CE and 2 ml L^−1^ of methanol with 5% (V/V) inoculum and incubated at 28°C and 150 rpm for 8 days. Optical density (OD_600_) and herbicide concentration were measured every 24 h to determine the bacterial cell density and degradation rate of CE.

The degradation rate was measured by high-performance liquid chromatography (HPLC). In brief, 10 ml of culture medium and 10 ml of dichloromethane were mixed in a 150 ml separatory funnel, and the lower organic phase was combined after three rounds of extraction by vigorous shaking. The organic phase was dried with N_2_, suspended in 10 ml acetonitrile, and filtered through a 0.22 μm nylon membrane. Then, a 20-μl sample was injected into the HPLC equipped with a Zorbax SB-C18 ODS Spherex column (4.6 × 250 mm, 5 μm, Agilent Technologies, Santa Clara, CA, USA) at 25°C; the mobile phase was acetonitrile (A): 0.05% acetic acid (B) at a flow rate of 1 ml/min, linear gradient 0–1 min 2% A; 1–10 min 2–70% A; 10–13 min 70–100% A; 13–13.5 min 100–2% A; and 13.5–15 min 2–0% A (Wang et al., 2016). CE was detected at 254 nm. The correlation coefficient (*R*^2^) for the standard curve was 0.9996.

To use sequencing methods to study the interactions and changes of MC-L1 during the biodegradation process, the bacterial pellet samples were collected by centrifuging at 80,000 rpm for 15 min according to the degradation curve and growth curve. In addition, we also collected an incapacitated consortium (marked NO) belonging to the same generation and then also performed a sequencing analysis.

### DNA Extraction

Total DNA was extracted using the E.Z.N.A.™ Soil DNA Kit (Omega Biotek, Inc., Norcross, GA, USA), and its concentration and purity were evaluated using a Nanodrop 2000 Spectrophotometer (Thermo Scientific, Wilmington, DE, USA). After quality control, the extracted DNA samples were stored at −80°C in preparation for sequencing (Chen et al., 2018).

### 16S rRNA Gene Sequencing and Analysis

The PCR amplifications were performed using primers 338F/806R targeting the V3–V4 region of the bacterial 16S rRNA gene (Su et al., 2018). The amplicons were merged on an Illumina MiSeq PE300 platform (Illumina Inc., San Diego, USA) following the standard protocols by Majorbio Bio-Pharm Technology Co. Ltd. (Shanghai, China). Raw sequences were filtered for quality using Quantitative Insights Into Microbial Ecology (QIIME, version 1.9.1) (Caporaso et al., 2012). After removing those low-quality sequences (quality scores < 20, length < 50 bp), operational taxonomic units (OTUs) were assigned from the reads using UPARSE (version 7.1 http://drive5.com/uparse/) at a 97% sequence similarity threshold (Edgar, 2013). The taxonomic identity of all phylotypes was then determined by the SILVA ribosomal RNA gene database project (Quast et al., 2013).

### Metagenomic Sequencing and Annotation

Metagenomic sequencing based on the NovaSeq 6000 platform (Illumina, USA) was completed by Majorbio, Inc. (Shanghai, China). Metagenomic assembly, contigs binning, gene prediction, and abundance analysis were performed according to a previous study (Zhang et al., 2020b). The gene catalog was translated to putative amino acid sequences, which were all extracted from the NCBI NR database, evolutionary genealogy of genes: Non-supervised Orthologous Groups (EggNOG, http://eggnog.embl.de/, version 4.5.1) and the Kyoto Encyclopedia of Genes and Genomes (KEGG, http://www.genome.jp/kegg/, version 94.2) (Ogata et al., 1999) databases with Diamond (http://www.diamondsearch.org/index.php, version 0.8.35) (*e*-value ≤ 1e^−5^). The original sequencing data have been uploaded to the NCBI database (accession number: PRJNA788073 for 16S rRNA high-throughput sequence, PRJNA788363 for metagenomic sequence).

### Correlation Network Analyses

Network analysis was performed to reveal the complex associations within microbial communities and identify potential keystone taxa for degradation (Zheng et al., 2021). Networks of different time points and incapacitated consortia were implemented on the I-Sanger platform using Networkx and visualized with Cytoscape 3.8.2 (Banerjee et al., 2016). Co-occurrence relationships were considered as stable if the Pearson's correlation coefficient was >0.5 and *P* < 0.05. All operational taxonomic units (OTUs) were analyzed; the color level of each node is displayed as a level. Nodes showing a high degree were considered keystone taxa (Berry and Widder, 2014).

To visualize the associations between species and function, we established a function-taxon correlation network to identify the functional clusters of bacterial taxa in MC-L1. The top 50 genera and top 50 functions in pathway level 3 were selected for the network constructions. Pearson's correlation coefficients used in the network analysis were more than 0.5 and the cutoff of *P*-values was 0.05. Finally, we also used Cytoscape 3.8.2 (Banerjee et al., 2016) for visualization. The taxon nodes tending to have high degrees were identified as degradation keystone taxa.

### Statistical Analyses

Degradation curve and growth analyses were performed using the GraphPad Prism 8 software (GraphPad, Inc., La Jolla, CA, USA). One-way analysis of variance (ANOVA) and Pearson's correlation analysis between CE residues and the OD_600_ were performed using the SPSS 25 software (SPSS, Inc., Chicago, IL, USA). Alpha diversity indices (within samples) were calculated using Mothur version 1.30.1 (Schloss and Westcott, 2011). Beta diversity (between samples) was visualized using principal coordinate analysis (PCoA) of the Bray Curtis distance metric. Permutational multivariate ANOVA (PERMANOVA) based on the Bray-Curtis similarity (Bray and Curtis, 1957) was also performed to identify variations among different groups with 999 permutations. Hierarchical clustering analysis of the dataset was performed using the Bray-Curtis distance at the genus level. Venn diagrams and composition diagrams of the sample microbiota were drawn in the Vegan package (version 2.4.3) using R (The R Project for Statistical Computing, Vienna, Austria). Differentially abundant bacterial genera among groups were determined using linear discriminant analysis (LDA) effect size (LEfSe), applying the all-against-all strategy with a threshold of 3 on the logarithmic LDA score for discriminative features (Segata et al., 2011). The Circos graph, multigroup comparison, and relative contribution analysis were performed using the Majorbio I-Sanger Cloud Platform (www.i-sanger.com).

## Results and Discussion

### Enrichment and CE Degradation of L1 Consortium

**Figure 1:**
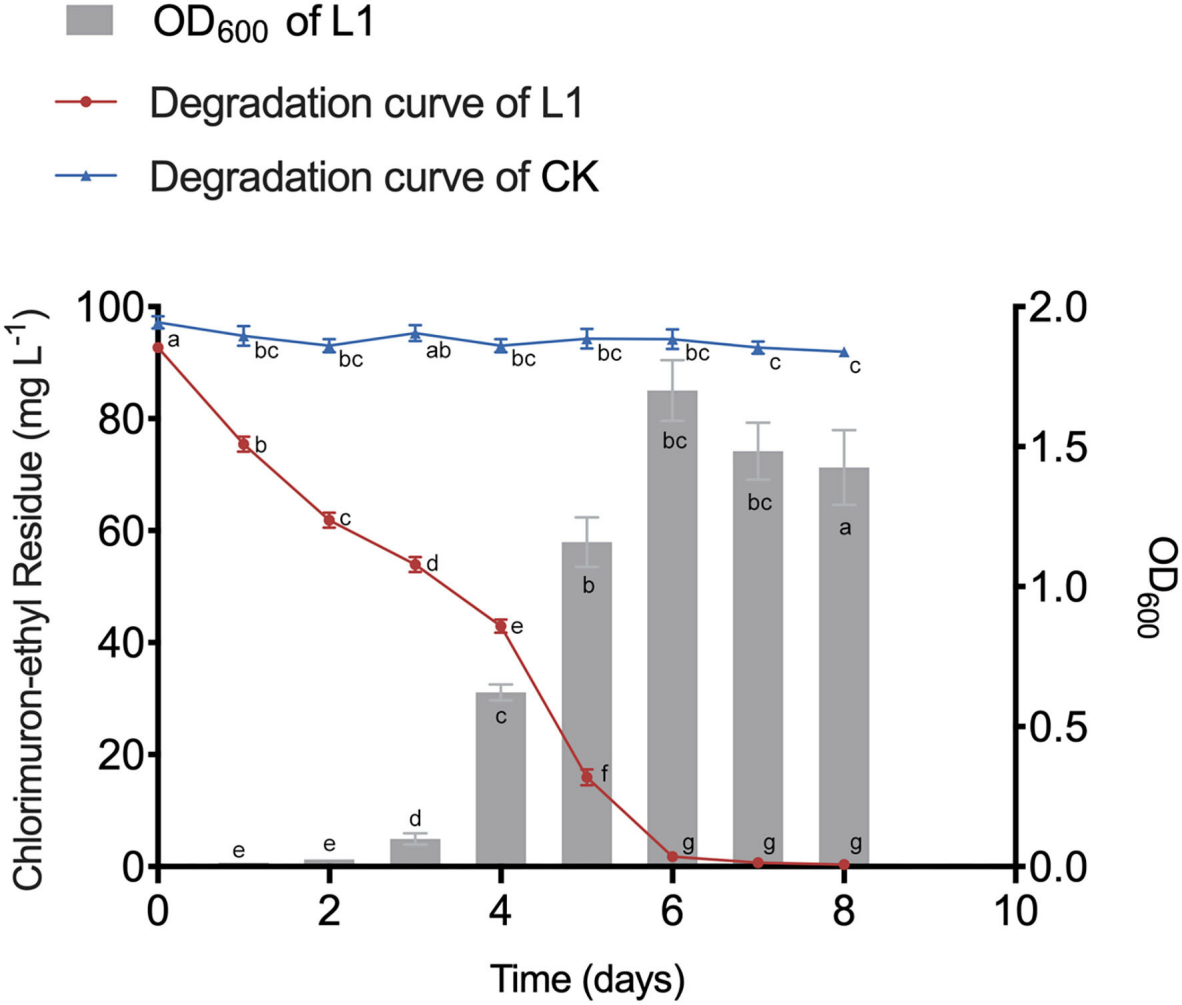
Growth dynamics of microbial consortia L1 (MC-L1) and its degradation curve to chlorimuron-ethyl.

After inoculation, CE was rapidly degraded without any lag period (Figure 1). MC-L1 almost completely degraded CE within 8 days (99.62%), with the degradation rate reaching 98.04% at 6 days (Figure 1). The degradation rate and tolerance to high contamination stress of MC-L1 were significantly better than the consortium reported previously (Li et al., 2017). This may be because the natural microbial consortium has evolved to have more complete co-metabolic networks, closer interactions, and stronger environmental adaptations (Kato et al., 2008). As shown in Figure 1, the OD_600_ increased rapidly from days 3 to 6 and then decreased slowly due to the depletion of carbon and energy materials after CE degradation. An extremely strong negative correlation was observed between the OD_600_ and CE residues (*R* = −0.95, *P* < 0.01), indicating that biomass is important for the efficient degradation of MC-L1. Therefore, corresponding to the pre-degradation, degradation, and post-degradation periods, consortium L1 was selected on days 1, 4, 5, and 7 for 16S rDNA and metagenomic sequencing.

### Overview of 16S rRNA Sequencing and Metagenomic Sequencing

After sequence quality control, 1,032,755 effective sequence reads with an average length of 419.24 bp were obtained from all samples using 16S rRNA high-throughput sequencing. These sequences were assigned to 78 OTUs with 97% similarity. The number of observed OTUs, library coverage, species richness, and diversity indices is shown in Supplementary Table S1.

Metagenomic sequencing generated 1.15 million contigs comprising 1.66 billion contigs bases (Supplementary Table S2). To identify the potential biological pathways of the genes, we used genes related to xenobiotic biodegradation and metabolism (XBM) in the KEGG to construct and re-annotated a new gene set. All analyses of the metagenomic data were based on this new gene set, in which 5,281 genes were mapped into five branches with 89 pathways. The “xenobiotics bio-degradation and metabolism” pathway belonged to level 3 and involved 21 different pathways, among which the most numerous pathways were related to the metabolism of benzene ring structures including “benzoate degradation,” “styrene degradation,” “xylene degradation,” “toluene degradation,” and “naphthalene degradation”. Some pathways were associated with the degradation of chlorinated substances, such as chlorocyclohexane and chlorobenzene degradation and chloroalkane and chloroalkene degradation. These results suggest that many pathways and genes in MC-L1 are related to the degradation of a wide range of pollutants.

### Richness and Diversity of CE-Degrading Consortium L1

**Figure 2:**
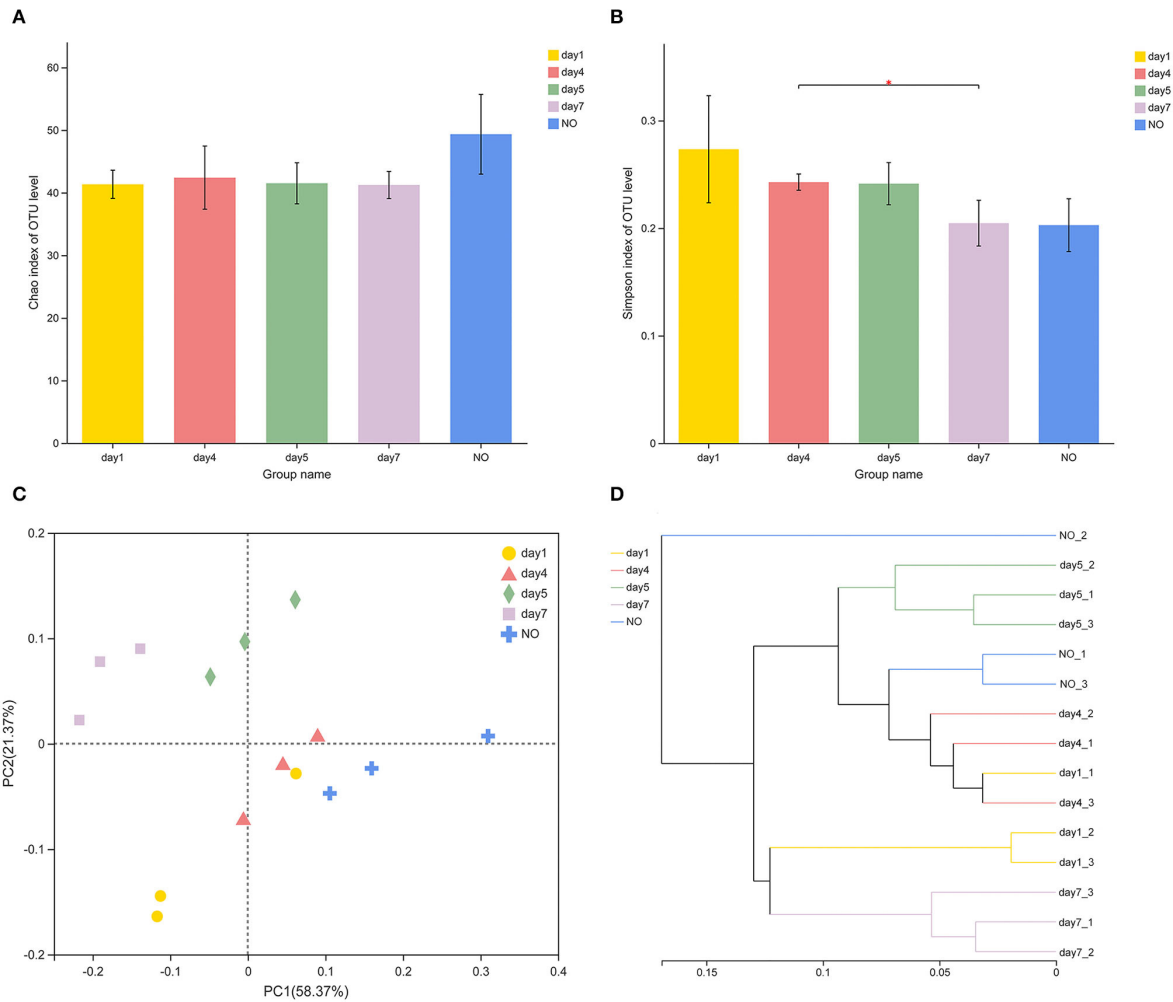
Variations in diversity and community structure of different consortium samples. **(A,B)** Variations of Chao1 and Simpson indexes *P < 0.05 (Student's *t*-test); **(C)** principal coordinate analysis based on Bray–Curtis distances; **(D)** hierarchical cluster analysis using the Ward's method at the genus level.

In terms of alpha-diversity, the Chao and Simpson indices of MC-L1 did not significantly differ among groups (Figures 2A,B), whereas the Simpson indices significantly differed between the consortia on days 4 and 7 based on Student's *t*-test (*P* = 0.04303). The diversity of MC-L1 was relatively stable; therefore, the degradation capacity was not determined by the overall diversity but rather by the abundance of specific taxa (Banerjee et al., 2016). PCoA analysis was used to illustrate differences in β-diversity (Figure 2C). Bacterial community structures were divided into five groups at the genus level. Furthermore, PERMANOVA analysis indicated a significant effect of time on the bacterial structure (*R*^2^ = 0.7844, *P* = 0.001). The hierarchical clustering tree showed significant phylogenetic differences among all groups (Figure 2D). Samples from the lower degradation rate at the beginning (day 1) and ending stages (day 7) of the degradation process clustered into one group based on the phylogenetic composition, whereas consortia at the rapid degradation stage between days 4 and 5 had a different microbiota. These results demonstrate that the time factor did not alter the overall diversity of species in the microbial community but changed the abundance of key species.

### Composition and Structure of the Microbial Consortium in Different Groups

**Figure 3:**
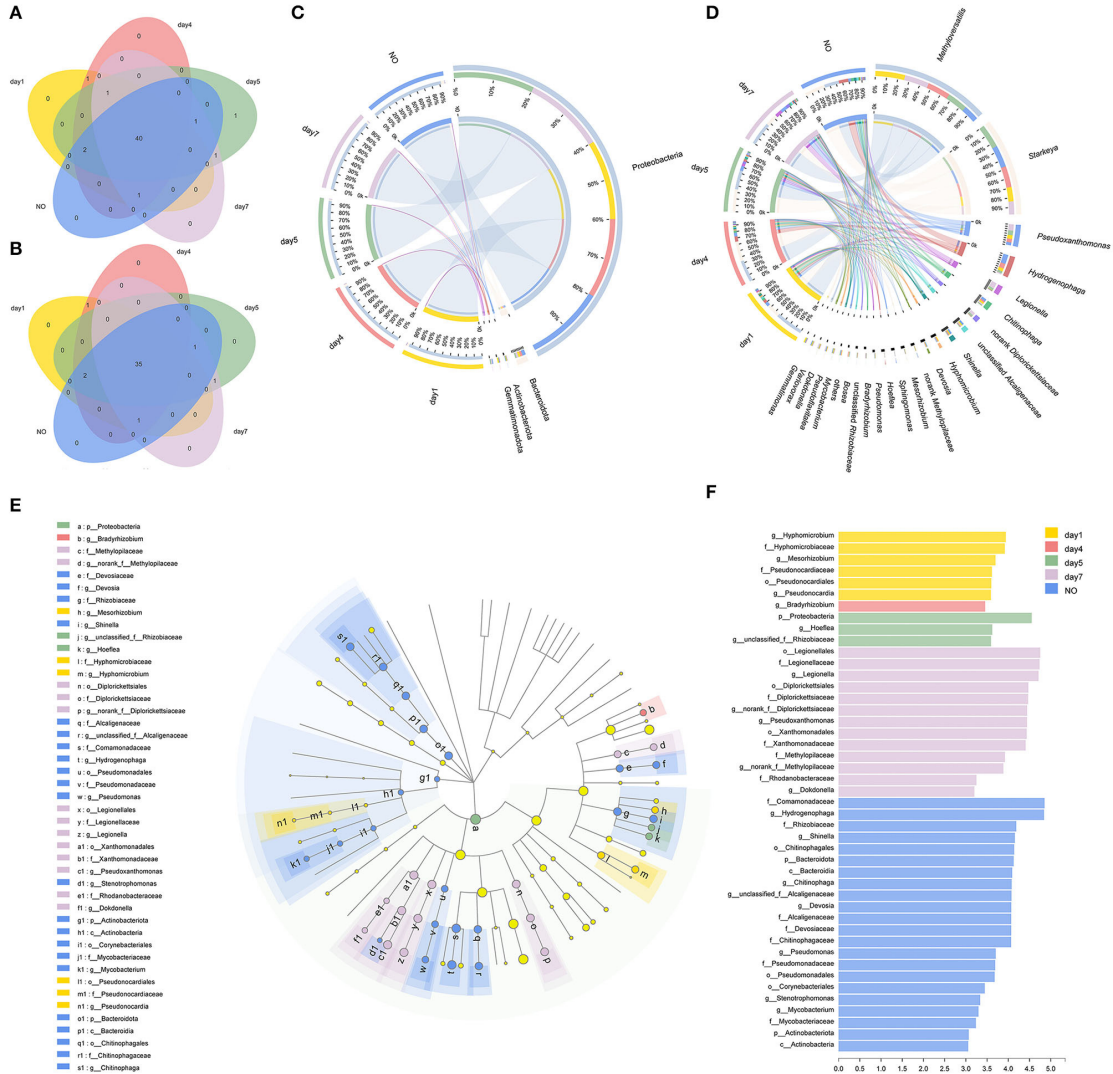
The evolution of bacterial community compositions among different time points and the incapacitated consortium. **(A,B)** Venn diagrams showed the shared and unique numbers of bacterial operational taxonomic units (OTUs) (at 97% identity) and genera among the five groups; **(C,D)** relationships between samples and phyla/genera are shown in Circos figures. Phylogenetic groups accounting for ≤ 1% of all classified sequences are summarized in the artificial group “others”; **(E,F)** phylogenetic cladogram of biomarker bacteria and indicator bacteria with linear discriminant analysis (LDA) scores of ≥3 in bacterial communities.

The Venn diagram showed unique and shared bacterial OTUs (Figure 3A) and genera (Figure 3B) in all five groups. The consortium on day 5 contained one unique OTU belonging to *Hyphomicrobium*, but no unique taxa were found at the genus level. Venn diagrams at the OTU and genus levels supported that there was no significant difference in the community composition between the consortium at different time points and incapacitated consortium. A previous study showed that using a single carbon source to enrich bacterial consortia results in the formation of a dense cross-feeding network, leading to collective interactions that simplify the interaction of the microbiome (Goldford et al., 2018). MC-L1 also formed relatively stable and close interactions under long-term CE stress as a single carbon source; therefore, the community composition did not change significantly.

The Circos figure illustrates that five phyla were identified in MC-L1 (Figure 3C), with Proteobacteria as the most dominant phylum (96.52%), particularly on day 5 (Kruskal–Wallis (K–W) *H* test, *P* < 0.05). The results were consistent with the report that Proteobacteria was the most abundant phylum in the resources of herbicide-degrading bacteria (Singh and Singh, 2016). At the genus level (Figure 3D), the bacterial communities were dominated by *Methyloversatilis* (34.31%, on days 1, 4, and 7) and *Starkeya* (28.60%, on day 5 and NO), followed by *Pseudoxanthomonas* (7.01%), *Hydrogenophaga* (6.83%), *Legionella* (3.47%), and *Chitinophaga* (3.12%). During degradation, *Starkeya* and *Hydrogenophaga* (K–W test, *P* < 0.05) first showed an increasing trend followed by a decreasing trend, and their abundance in the NO group was relatively high. The genus *Pseudoxanthomonas* showed an increasing trend, but its abundance in the NO group was the lowest (K–W test, *P* < 0.05), and the genus *Legionella* was set to increase (K–W test, *P* < 0.05). Significant temporal variations of bacterial community structure at the genus level were observed among different groups. Although none of these dominant genera have been reported to degrade CE, their consortium showed high degradation potential, possibly *via* cooperative catabolism, where one strain transforms the herbicide into products that are used by another strain (Ellegaard-Jensen et al., 2014).

Furthermore, we evaluated the presence of different bacterial genera by LEfSe analysis (*P* < 0.05, LDA score >3) and identified 45 taxonomic clades with different abundances and LDA scores higher than 3.0 (Figures 3E,F). We found that 6, 1, 3, 13, and 22 taxa were significantly enriched in each group, with the NO sample showing the largest number of significantly enriched microbes, such as Bacteroidota (from phylum to genus), Actinobacteria (from phylum to genus), and Pseudomonas (from order to genus). Taken together, NO greatly differed in community structure from the other consortium samples. Thus, the loss of degradation capacity can occur even in relatively stable consortia; additionally, although the overall community composition did not change, overgrowth and/or growth failure of certain members can result in structural changes that may affect co-metabolic processes (Kato et al., 2008).

### Microbiome Function Annotation Based on Metagenomic Sequencing

The relative abundance of metagenomic next-generation sequencing (mNGS) and 16S rRNA sequencing showed that MC-L1 was clearly dominated by Rhizobiales (Supplementary Figure S1A). Other orders present in the consortium were Rhodocyclales, Burkholderiales, and Xanthomonadales. In addition, some orders such as Sphingomonadales and Legionellales showed a lower relative abundance in mNGS than in 16S rRNA sequencing. The main difference between the mNGS and 16S rRNA sequencing relative order abundance was found in Rhodocyclales, which was relatively abundant according to the mNGS results (37.90%) but absent from the 16S rRNA sequencing results. This result was explained by differences in sequencing principles and taxonomic identification databases (SILVA database/NCBI NR database). In addition, the genetic similarity of some species was high, which may lead to errors in phylogenetic analyses, such as *Sphingomonas* and *Sphingobium* (Zhao et al., 2017). Overall, the compositions of the four main orders in mNGS (93.89%) and 16S rRNA sequencing (89.06%) were similar, indicating consistency between the two sequencing results.

The results of PCoA analysis of KEGG at level 3 of “XBM” showed significant differences between the NO group and other groups (Supplementary Figure S1B). PERMANOVA confirmed these significant differences (*R*^2^ = 0.79897, *P* = 0.002 in KEGG), enabling the analysis and prediction of functional bacteria with degradation ability. Based on this, the functional analysis should focus not only on the differences between the degradation consortium at different time points but also on samples without degradation functions.

**Figure 4:**
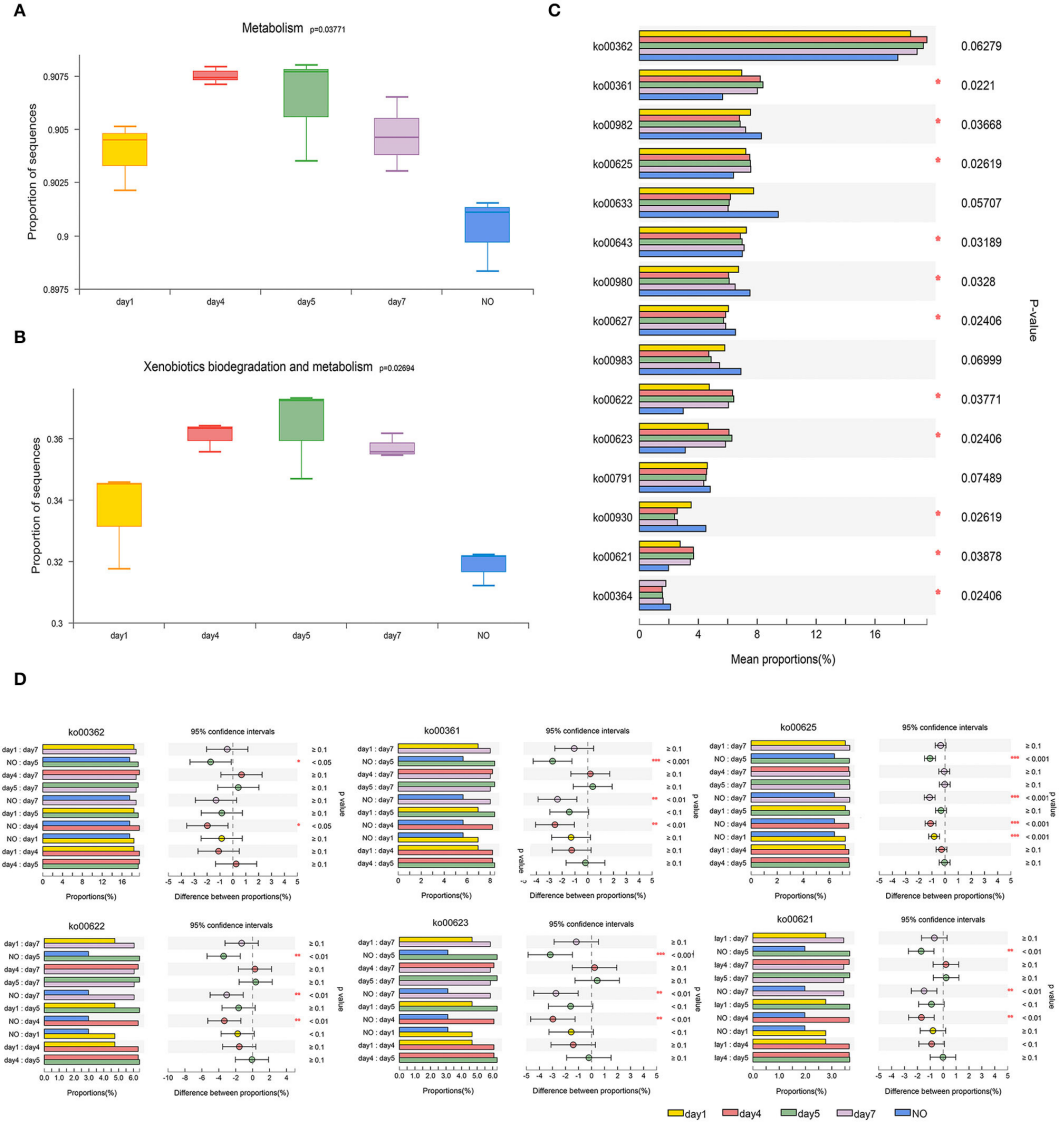
The comparison of bacterial community functions analyzed at level 1, level 2, and level 3 between different groups. **(A)** and **(B)** Comparison of the Kyoto Encyclopedia of Genes and Genomes (KEGG) functions at level 1 related to “Metabolism” and level 2 related to “Xenobiotics Biodegradation and Metabolism”; **(C)** The Kruskal–Wallis (K–W) test of the top 15 abundance KEGG pathways at level 3 in the category of “Xenobiotics Biodegradation and Metabolism”; **(D)** Tukey-Kramer test using *post-hoc* analysis of six KEGG pathways. *P < 0.05, **P < 0.01, ***P < 0.001.

As shown in Figure 4, a multigroup comparison based on the K–W test revealed significant differences among the five groups for levels 1, 2, and 3. In both the KEGG categories of metabolism (Figure 4A) and “XBM” (Figure 4B), functional genes tend to first increase and then decrease during degradation. Particularly, samples on day 5 showed the most significant enrichment, whereas NO exhibited the lowest enrichment (K–W test, *P* < 0.05).

At level 3 in the “XBM” category, we analyzed the top 15 KEGG pathways (Figure 4C), among which 11 pathways exhibited significant differences. Among the 15 pathways, six were selected for further analysis, namely, ko00362 (benzoate degradation), ko00361 (chlorocyclohexane and chlorobenzene degradation), ko00625 (chloroalkane and chloroalkene degradation), ko00622 (xylene degradation), ko00623 (toluene degradation), and ko00621 (dioxin degradation) because the functional genes of these six pathways in the NO group were significantly lower than those in samples at other time points. The abundance of functional genes was highly consistent with the trend of the degradation curve (Figure 4D).

The six pathways mentioned above involve degradation of the benzene ring structure and chloride, which agrees with previous studies on the degradation of CE *via* urea bridge cleavage, de-esterification, oxidation, cyclization, and cleavage of the N–C bond of the sulfonylurea bridge and pyrimidine ring (Li et al., 2016). In addition, “toluene degradation,” “chlorocyclohexane degradation, ” and “chlorobenzene degradation” were reported to respond to CE in *Rhodococcus erythropolis* D310-1 (Cheng et al., 2018). The six pathways were tested using the Tukey-Kramer *post-hoc* analysis. Although ko00362 was not significant in the multigroup comparison, we found significant differences in ko00362 between NO and the samples on days 4 and 5. Four of the six pathways, ko00361, ko00622, ko00623, and ko00621, showed remarkable differences between NO and the samples on days 4, 5, and 7, respectively. In ko00625, NO was extremely significantly different from all other groups (*P* < 0.001). The decrease in functional genes involved in these metabolic pathways in NO may lead to loss of the degradation ability of MC-L1.

### Linking the Potential Degrading Taxonomic and Functional Properties

**Figure 5:**
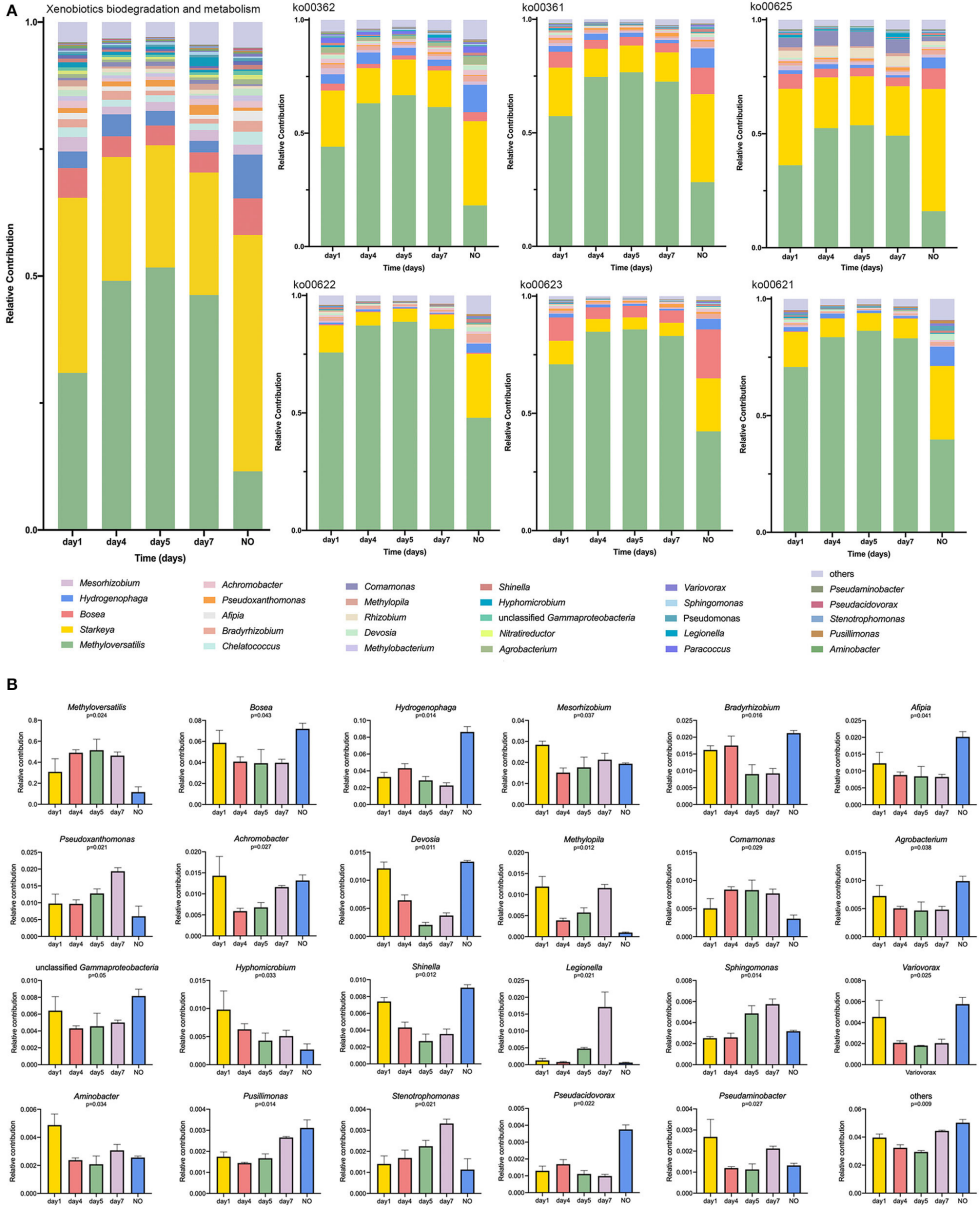
**(A)** Relative contribution of different taxa at genus level to identified degradation-enriched functional attributes in different groups; **(B)** The K–W *H* test at level 3 in the category of “Xenobiotics Biodegradation and Metabolism.”

To determine the relationship between potential degrading taxonomic and functional properties, we performed a relative contribution analysis at level 3 in the category of “XBM” and the six pathways (Figure 5A). *Methyloversatilis* was the main contributor among samples at different time points, but its contribution to NO was the lowest among the five groups (Figure 5A). The contribution of *Methyloversatilis* first increased and then decreased during degradation. This trend was not only reflected at these two levels but also was consistent with our results on the metabolic pathways and community structure of 16S rRNA sequencing. The relative contribution of *Starkeya* was highest in NO, particularly in ko00625, which is closely related to CE degradation. Thus, loss of the degradation ability of NO may be related to changes in the community structure, that is, a decrease in *Methyloversatilis* and an increase in *Starkeya*. Similarly, the contribution of taxa belonging to *Bosea* declined. The contribution of this genus was the highest on the first day and then decreased but was significantly higher in NO than in other samples, particularly in ko00361, ko00625, and ko00623. Interestingly, *Bosea* was nearly undetectable in ko00622 and ko00621, demonstrating that compared with other dominant strains, some pathways were lacking (Figure 5A). Additionally, some taxa showed higher contributions to NO than in other samples, such as *Afipia, Agrobacterium, Shinella*, and *Pseudacidovorax*, indicating that structural imbalances of these dominant genera can lead to a reduction in degrading bacteria and eventual loss of their degradation ability.

**Figure 6:**
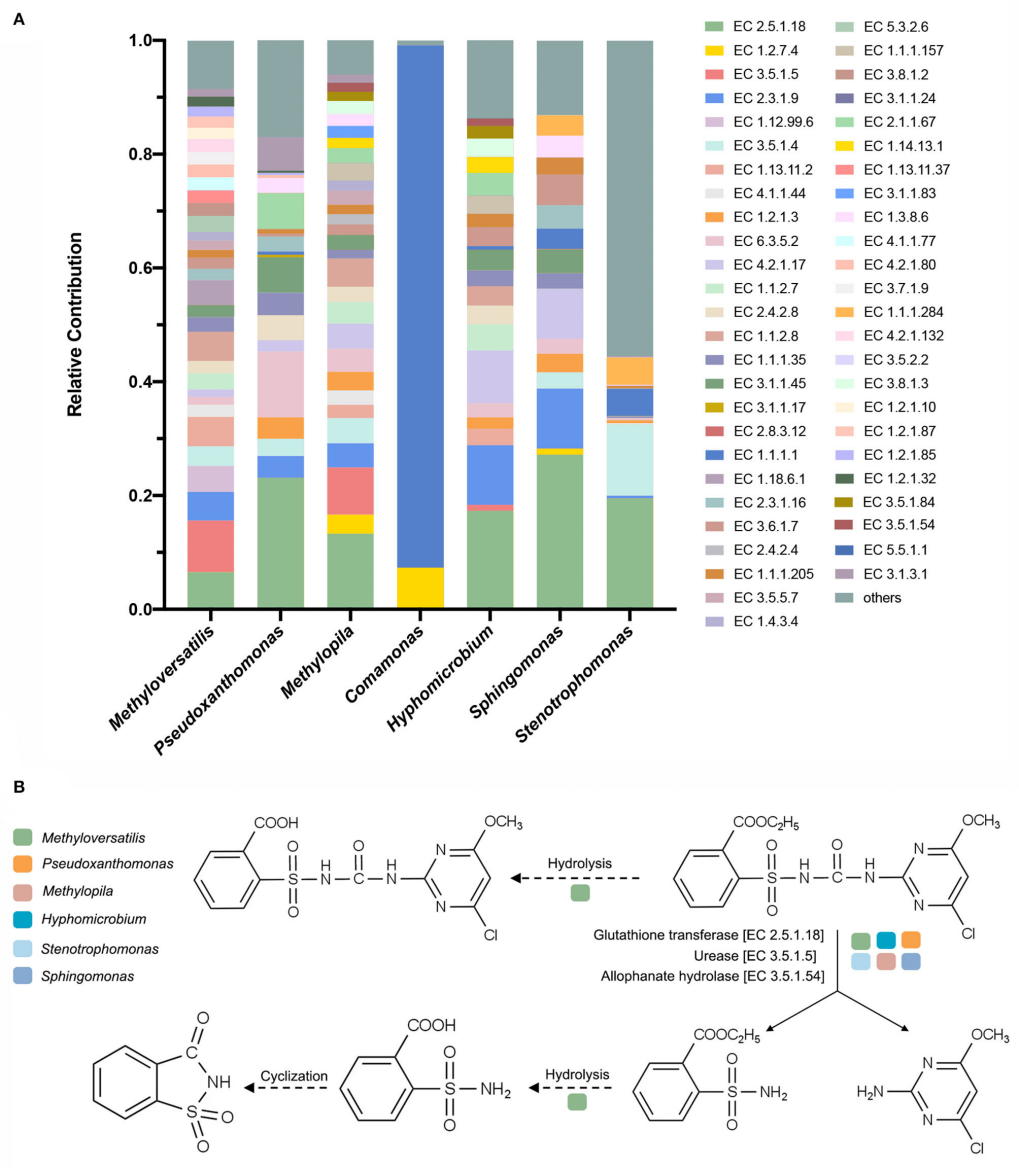
**(A)** Analysis of the functional contribution of seven possible degrading genera at the enzyme level; **(B)** proposed degradation pathway of chlorimuron-ethyl by MC-L1. Solid lines indicate the enzymes and pathways present in the MC-L1; dashed lines indicate the inferred pathways.

According to the K–W test (Figure 5B), the genera that may play important roles in CE degradation by MC-L1 were predicted. The following seven genera showed a high contribution to the degradation process but were low in NO: *Methyloversatilis, Comamonas, Pseudoxanthomonas, Methylopila, Sphingomonas, Hyphomicrobium*, and *Stenotrophomonas*. Analysis of the functional contribution of these genera at the enzyme level (top 50 enzymes) showed that *Methyloversatilis* contained the largest number of enzymes (32), whereas *Comamonas* contained the smallest number (2) (Figure 6A). *Methyloversatilis* likely has a wide range of metabolic functions and acts as the main functional strain during degradation. An analysis of the reactions catalyzed by the annotated enzymes, i.e., glutathione transferase [EC 2.5.1.18], urease [EC 3.5.1.5], and allophanate hydrolase [EC 3.5.1.54], was predicted to be relevant to CE degradation. All three enzymes can catalyze the cleavage of the CE carbamide bridge to produce 2-amino-4-chloro-6-methoxypyrimidine and ethyl o-sulfonamide benzoate, which is consistent with the previously reported degradation pathway of CE (Ma et al., 2009; Sharma et al., 2012; Zhang et al., 2020c). In terms of their functional contributions, glutathione transferase [EC 2.5.1.18] was annotated as the highest relative contributor in the six genera, except for *Comamonas*. Knockout experiments of *Klebsiella jilinsis* 2N3 confirmed the degradation capacity of glutathione transferase for CE (Zhang et al., 2020c), and comparative genome analysis of *Sphingomonas* showed that this bacterium produces amidohydrolase, which may catalyze the hydrolysis of the amide bond of CE (Cheng et al., 2019). This enzyme may play a dominant role in the degradation of CE by MC-L1, and *Methyloversatilis, Pseudoxanthomonas, Methylopila, Sphingomonas, Hyphomicrobium*, and *Stenotrophomonas*, which contain this enzyme, are the main degrading genera. In addition, allophanate hydrolase [EC 3.5.1.54] with a low relative contribution was annotated in *Methylopila* and *Hyphomicrobium*, and urease [EC 3.5.1.5] with a high relative contribution was annotated in *Methyloversatilis, Methylopila*, and *Hyphomicrobium*. The coexistence of all three enzymes in *Methylopila* and *Hyphomicrobium* suggested that these two genera had high degradation potentials. Although these three degradation-related enzymes have not been annotated in *Comamonas*, the high presence of alcohol dehydrogenase [EC1.1.1.1] suggests that this genus plays an important role in converting downstream degradation products for the energy supply. Additionally, previous studies showed that *Methyloversatilis* promotes the cleavage of ester bonds, which may be related to the production of 2-[[(4-chloro-6-methoxy-2-pyrimidinyl) carbamoyl] sulfamoyl] benzoic acid and 2-carboxy phenylsulfamide during the degradation of CE (Cai et al., 2011).

Although there are few reports of CE degradation by these six genera, substrate analyses suggested that they have a high potential for organic compound degradation. For example, *Methylopila* sp. can degrade a variety of sulfonylurea herbicides, such as metsulfuron-methyl and bensulfuron-methyl (Huang et al., 2007), and *Hyphomicrobium* sp. can degrade dichloromethane, methyl chloride, methamidophos, dimethyl sulfide, methanol, and polycyclic aromatic hydrocarbons (Yoshikawa et al., 2017; Hayoun et al., 2020; Jin et al., 2022). *Pseudoxanthomonas spadix* BD-a59 can metabolize all six BTEX compounds (Lee et al., 2012), and *Stenotrophomonas* sp. can metabolize aromatic compounds and highly chlorinated polychlorinated biphenyl congeners (Gao et al., 2013; Horváthová et al., 2018; Li et al., 2021). Additionally, *Stenotrophomonas maltophilia* D310-3 degraded 89% of 50.21 mg L^−1^ CE in 6 days (Zang et al., 2016). The genome of *Methyloversatilis universalis* FAM 5^T^ contained genes not only for the uptake and utilization of hydrogen and nitrogen compounds for energy metabolism but also for the utilization of cyanate, glycerol, long-chain amines, aromatic compounds, alkanesulfonates, alkylnitronate, phenols, acetone, urea, and methane sulfonic acid (meta-cleavage pathway) (Kittichotirat et al., 2011). *Sphingomonas* can mineralize chloroacetanilide and substitute urea herbicides, as well as aromatic compounds, such as phenol and chloramphenicol (Sørensen et al., 2013; Ruan et al., 2018; Cheng et al., 2019; Zhang et al., 2020a). Notably, *Sphingobium, Sphingomonas, Hyphomicrobium, Bosea*, and *Afipia* were enriched in the chloramphenicol-degrading bacterial consortium, indicating that these genera contain important functional genes and special co-metabolic networks for degrading benzene ring structures and chlorides (Zhang et al., 2020a). Network analysis can help reveal these non-random interactions and define the functions of the genera (Fuhrman, 2009).

In summary, we identified six functional genera in MC-L1 that are directly involved in CE degradation and identified the possible reactions involved in each genus (Figure 6B). The main reaction steps are as follows: glutathione transferase [EC 2.5.1.18], urease [EC 3.5.1.5], and allophanate hydrolase [EC 3.5.1.54] in *Methyloversatilis, Pseudoxanthomonas, Methylopila, Sphingomonas, Hyphomicrobium*, and *Stenotrophomonas* directly catalyzed cleavage of the CE carbamide bridge to produce 2-amino-4-chloro-6-methoxypyrimidine and ethyl o-sulfonamide benzoate. Some enzymes in *Methyloversatilis* may then catalyze the hydrolysis of the ethyl ester on the benzene ring by undergoing a hydrolysis reaction to produce o-sulfonamide benzoic acid, which in turn undergoes a cyclization reaction to form o-sulfonate benzoic imide.

### Correlation Network Analysis of Bacterial Consortium L1

**Figure 7:**
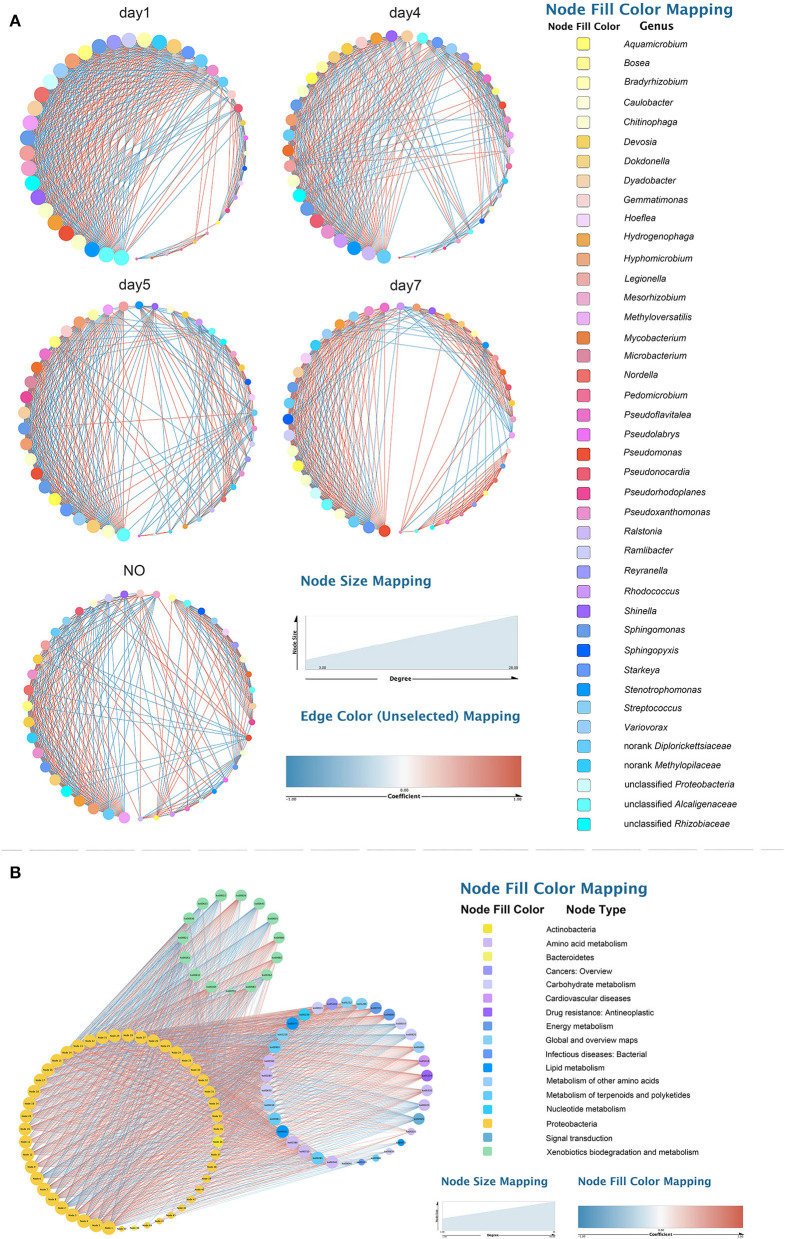
Networks of the microbial consortium in different time points and incapable consortium **(A)** co-occurrence network analysis; **(B)** species/function correlation network. Node size is proportional to node degree. Node colors represent various phylogenetic genera. Red lines indicate positive interactions, and blue lines indicate negative interactions.

To explore changes in the co-occurrence patterns of the consortium at different time points, five complex networks were constructed for different groups using OTU data (Figure 7A). As shown in Table 1, although all five networks showed close interactions, the complexity of the networks decreased with degradation, whereas the NO network was the simplest. This may be because at the start of degradation, microorganisms can use methanol to rapidly increase biomass and degrade CE through co-metabolism; however, as the substrate concentration decreases, the interactions gradually weaken, which is consistent with the pattern of microbial carbon utilization, that is, simple alcohols are utilized before the complex organic matter is used. The number of edges with positive and negative correlations was similar across the five networks; however, the day 7 group clearly showed more positive correlation edges than negative (Table 1), indicating a cooperative relationship of mutualism or commensalism. At the end of degradation, CE is essentially catabolized into bioavailable metabolites, and the bacteria responsible for downstream biodegradation may perform cross-feeding by exchanging metabolic products or using the metabolites of other bacteria to grow and flourish (Woyke et al., 2006; Faust and Raes, 2012). The transitivity and average degree of the network were highest on day 1 and lowest in NO; however, the average shortest path length of the network was highest in NO and lowest on day 1. These results suggest that the apparent reduction in bacterial network complexity was closely associated with the loss of degradation function, indicating a reduction in interactions between the bacterial communities.

**Table 1:** Attribute table of five co-occurrence networks.

**Sample**	**Node**	**Edge**	**Positive edge**	**Negative edge**	**Transitivity**	**Avg. shortest path length**	**Avg. degree**	**Keystone numbers**
Day 1	45	457	226	231	0.90402534	1.96969697	20.31	28
Day 4	45	384	190	194	0.81281407	2.22828283	17.07	26
Day 5	46	351	176	175	0.84092098	2.29178744	15.26	19
Day 7	44	321	218	103	0.85828877	2.23890063	14.59	10
No	44	311	163	148	0.80847688	2.36363636	14.14	6

Based on the high-degree node (degree ≥20), 28, 26, 19, 10, and 6 keystone taxa were selected from the five complex networks. The number of keystone taxa in NO and day 7 groups was far smaller than that in the early and middle degradation stages. These results indicate that the abundance of CE in the early degradation stages created many trophic levels or resource cascades so that some keystone taxa cooperate to achieve co-metabolism. In these keystone taxa, 10 OTUs belonging to six genera appeared in four groups, among which three genera, namely, *Chitinophaga, Starkeya*, and unclassified *Alcaligenaceae*, were dominant and appeared in networks of four time points, thus participating in the whole process of degradation (Supplementary Table S3). In the keystone taxa, eight OTUs belonging to seven genera appeared in the three groups, whereas only five genera appeared in the consortium with degrading ability. These five genera, namely, *Variovorax, Sphingomonas, Pseudomonas, Dyadobacter*, and *Aquamicrobium*, accounted for a small proportion of the community composition. Keystone species are commonly considered to exert a large effect on the ecosystem, which may not be proportional to their abundance because their impact on the community is shaped by their interactions with other members (Berry and Widder, 2014). Specifically, *Sphingomonas* was related to not only the degradation according to KEGG functional analysis but also a keystone taxon in the degradation process based on the results of network analysis. This may be because *Sphingomonas* can catalyze the amide bond hydrolysis reaction, providing a substrate for downstream microorganisms. In addition, there were 4, 4, 2, and 1 unique OTUs in the five networks, respectively.

In the function-taxon correlation network, there were 47 taxon nodes, 49 functional nodes, and 1,593 edges, of which 965 were positively correlated and 628 were negatively correlated (Figure 7B). Generally, close ecological interactions, functional differentiation, and metabolite exchange in consortia may allow coexisting species to cycle nutrients efficiently, improve the overall resource utilization efficiency, and acquire robust tolerance and resilience to environmental disturbances (Kato et al., 2008; Bernstein and Carlson, 2012). The attributes of the taxon and function nodes in the networks of all samples are shown in Supplementary Table S4. Among all functional nodes, 15 nodes associated with “XBM” ranked first, followed by 8 nodes related to “amino acid metabolism”. Among all taxon nodes, seven nodes had a degree distribution higher than 40; three of these were related to “amino acid metabolism”. The degree of the six pathways was 33–37. This result indicates that the performance of functions related to CE degradation by MC-L1 requires the collaboration of multiple strains. The synergistic metabolism among strains of the consortium resulted in a higher degradation efficiency compared with that of a single bacterium.

Among all taxon nodes, there were 29 nodes with a degree distribution higher than 40; the highest degree was 46. The degree of correlation between these nodes and “XBM” was 14. *Methyloversatilis* was not only a keystone taxon in the co-occurrence network but also extremely important in the function-taxon correlation network. In addition, as a dominant genus, *Methyloversatilis* was identified in K–W test analysis as a possible degrading genus. Interestingly, some bacteria such as *Afipia, Agrobacterium, Shinella*, and *Pseudacidovorax* which have not been predicted to be associated with degradation, showed a high degree. This indicates that the degradation of CE in MC-L1 requires not only “functional bacteria” with degradability (showing a direct effect on hydrocarbons) but also “auxiliary bacteria” without degradability but that promote/inhibit the degradation process *via* synergistic growth and metabolism. The abundance of “auxiliary bacteria” must be controlled within a suitable range. *Pseudoxanthomonas, Stenotrophomonas*, and *Sphingobium* (*Sphingomonas*), which were considered degrading bacterial strains according to KEGG analysis, showed had a high degree, specifically in the degree centrality, closeness, and betweenness centrality.

In summary, network analysis provides information on co-occurrence and functions, providing a foundation for analyzing the interactions between bacteria. Further studies should focus on the isolation and cultivation of these strains, based on which the construction, characterization, and modeling of artificial synthetic microbial communities can be performed to further improve the degradation efficiency and range of degradable substrates of the new synthetic consortium for application in the remediation of herbicide contamination.

## Conclusion

In this study, we obtained the novel microbial consortium L1 using enrichment culture. The microbial consortium L1 degraded 98.04% of 100 mg L^−1^ CE within 6 days, which is superior to all CE consortia reported to date. In addition, 16S rRNA high-throughput sequencing and metagenomic sequencing were performed to comprehensively characterize changes in the diversity and structural and functional interactions of microbial consortium L1 during degradation and to predict the metabolic enzymes, pathways, and degrading genera of the chlorimuron-degrading consortium. These findings provide insight for further exploration of new microbial resources that can degrade sulfonylurea herbicides and a theoretical basis for the rational design of optimized artificial consortium models to investigate the mechanisms of microbial community formation and connection between the microbial community structure and ecological functions.

## Data Availability Statement

The datasets presented in this study can be found in online repositories. The name of the repository and accession numbers can be found below: NCBI Sequence Read Archive (SRA); PRJNA788363 and PRJNA788073.

## Author Contributions

XiaL performed the experiments, analyzed the data, and wrote the manuscript. CL, YD, and ZY performed the experiments. XinL, XW, and ZS assisted the manuscript checking. WG, XuL, and TL helped to modify the graphs. MX provided assistance and guidance throughout the research. HZ designed the experiments and revised the manuscript. All authors contributed to the article and approved the submitted version.

## Funding

This study was supported by the Strategic Priority Research Program of the Chinese Academy of Sciences (Grant No. XDA28010503), the Science and Technology Plan Projects of Shenyang City (20-203-5-57), the National Natural Science Foundation of China (31670515), and the PhD Research Startup Foundation of Liaoning Province (2020-BS-275).

## Conflict of Interest

The authors declare that the research was conducted in the absence of any commercial or financial relationships that could be construed as a potential conflict of interest.

## Publisher's Note

All claims expressed in this article are solely those of the authors and do not necessarily represent those of their affiliated organizations, or those of the publisher, the editors and the reviewers. Any product that may be evaluated in this article, or claim that may be made by its manufacturer, is not guaranteed or endorsed by the publisher.
