# Supplementary material for: Characterizing the Microbial Consortium L1 Capable of Efficiently Degrading Chlorimuron-Ethyl via Metagenome Combining 16S rDNA Sequencing
Source: Front Microbiol. 2022 Jun 23;13:912312. doi: 10.3389/fmicb.2022.912312 (PMC9260513; doi:10.3389/fmicb.2022.912312)
Supplement: Supplementary file 1 [file Table_1.DOCX]

Supplementary Material

**Supplementary Table 1.** Statistics of the trimmed sequences and alpha diversity of the bacterial communities.

| **Sample ID** | **Sequences** | **Bases (bp)** | **Average length (bp)** | **Coverage** | **Ace** | **Chao** | **Shannon** | **Simpson** |
| --- | --- | --- | --- | --- | --- | --- | --- | --- |
| day 1 | 70726 | 29723723 | 420.25 | 0.99993 | 41.8464±1.6969 | 41.3333±1.8409 | 1.9435±0.0948 | 0.2733±0.0407 |
| day4 | 67608.6667 | 28276101.6667 | 418.21 | 0.99989 | 45.2945±5.7315 | 42.4±4.1215 | 1.9488±0.0178 | 0.2427±0.0062 |
| day5 | 65701 | 27448834.6667 | 417.87 | 0.99995 | 41.7064±2.7903 | 41.5±2.6771 | 1.9872±0.0420 | 0.2413±0.0160 |
| day7 | 71757.6667 | 30284571 | 422.05 | 0.99994 | 41.8425±1.8826 | 41.2222±1.7708 | 2.1158±0.0792 | 0.2046±0.0174 |
| No | 68458.3333 | 28596439.6667 | 417.80 | 0.99981 | 68.1299±21.7426 | 49.3333±5.2015 | 2.0842±0.0724 | 0.2027±0.0201 |
| Total | 1032755 | 432989010 | 419.23 | - | - | - | - | - |
