# Supplementary material for: Characterizing the Microbial Consortium L1 Capable of Efficiently Degrading Chlorimuron-Ethyl via Metagenome Combining 16S rDNA Sequencing
Source: Front Microbiol. 2022 Jun 23;13:912312. doi: 10.3389/fmicb.2022.912312 (PMC9260513; doi:10.3389/fmicb.2022.912312)
Supplement: Supplementary file 2 [file Table_2.DOCX]

Supplementary Material

**Supplementary Table 2.** Statistics of raw data and clean data of metagenome sequencing.

| **Sample ID** | **Raw reads** | **Total bases (bp)** | **Clean reads** | **Clean bases(bp)** | **Percent in raw reads (%)** | **Contigs** | **Contigs bases (bp)** | **N50 (bp)** | **N90 (bp)** |
| --- | --- | --- | --- | --- | --- | --- | --- | --- | --- |
| day1 | 55591099.33 | 8394255999 | 54795356.67 | 8269460170 | 98.56% | 75673 | 122918926.3 | 4755 | 514 |
| day4 | 54635258 | 8249923958 | 54042235.33 | 8150793256 | 98.91% | 77631 | 103616134 | 2562 | 477 |
| day5 | 53504839.33 | 8079230739 | 52885478 | 7974376896 | 98.84% | 82112 | 103234318 | 2322 | 449 |
| day7 | 55133085.33 | 8325095885 | 54589226.67 | 8236140941 | 99.01% | 79366 | 125919729.7 | 4202 | 521 |
| No | 55092483.33 | 8318964983 | 54162338.67 | 8171057924 | 98.29% | 68410 | 98608755 | 5221 | 454 |
