# Supplementary figures and images for: Characterizing the Microbial Consortium L1 Capable of Efficiently Degrading Chlorimuron-Ethyl via Metagenome Combining 16S rDNA Sequencing
Source: Front Microbiol. 2022 Jun 23;13:912312. doi: 10.3389/fmicb.2022.912312 (PMC9260513; doi:10.3389/fmicb.2022.912312)

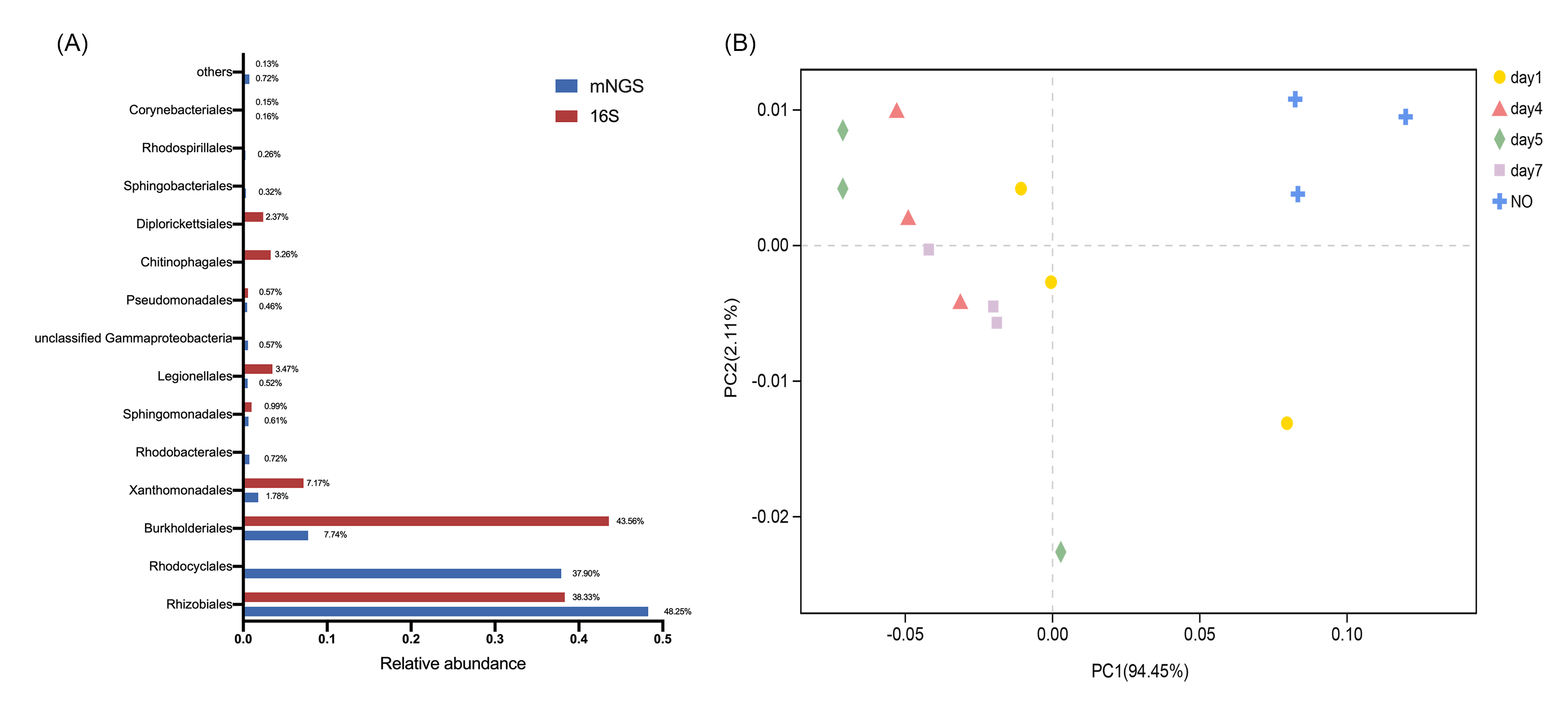

Supplement: Supplementary file 5 [file Image_1.TIF]
